# Duration of residence and psychotropic drug use in recently settled refugees in Sweden - a register-based study

**DOI:** 10.1186/s12939-014-0122-2

**Published:** 2014-12-20

**Authors:** Maria Brendler-Lindqvist, Marie Norredam, Anders Hjern

**Affiliations:** Copenhagen School of Medicine, University of Copenhagen, Copenhagen, Denmark; Danish Research Centre for Migration, Ethnicity and Health, Section of Health Service Research, Department of Public Health, Faculty of Health Sciences, University of Copenhagen, Copenhagen, Denmark; Section of Immigrant Medicine, Department of Infectious Diseases, Copenhagen University Hospital, Hvidovre, Denmark; Clinical Epidemiology, Department of Medicine, Karolinska Institutet, Stockholm, Sweden; Centre for Health Equity Studies, Karolinska Institutet/Stockholm University, 106 91 Stockholm, Sweden

**Keywords:** Refugee, Migration, Mental health, Psychotropic drugs, Acculturation, Access

## Abstract

**Introduction:**

Recently settled refugee populations have consistently been reported to have high rates of mental health problems, particularly Post-traumatic stress disorder, depression, and anxiety disorders. The aim of this study was to investigate psychotropic drug use among young adult refugees according to duration of residence during the first 10 years in Sweden.

**Methods:**

Cross-sectional register study of a national cohort of 43 403 refugees and their families (23–35 years old) from Iraq, Iran, Eritrea, Ethiopia, Somalia and Afghanistan and a comparison population of 1.1 million Swedish-born residents. Logistic regression was used to assess the association between duration of residence in Sweden and the dispensing of at least one psychotropic medication during 2009 in four categories (any drug, neuroleptics, antidepressants and anxiolytics/hypnotics), adjusting for age, gender and domicile.

**Results:**

Rates of dispensed psychotropic drugs among recently settled refugees were low, compared to the Swedish-born, with an increase with duration of residence. For refugee men and women from Iraq/Iran who had resided for 0–3 years the adjusted ORs compared to Swedish natives, were 0.83 (95% CI 0.77-0.90) and 0.48 (0.44-0.53) respectively; for men and women from the Horn of Africa the ORs were 0.50 (0.42-0.61) and 0.36 (0.30-0.41) respectively. After 7–10 years of residence, the ORs in these refugee groups approached the Swedish comparison population. Refugees from Afghanistan presented ORs similar to the Swedish-born, with no consistent trend by duration of residence. Women from the Horn of Africa and Iraq/Iran consumed less psychotropic drugs compared with men from these regions of origin, relative to the Swedish-born (p < 0.01). The ORs for dispensed neuroleptics were similar between the different refugee study groups, while the ORs for dispensed antidepressants differed fourfold between the group with the lowest (Horn of Africa) and the highest (Afghanistan).

**Conclusion:**

The rates of dispensed psychotropic drugs in the newly settled refugee populations in this study were low, with an increase with longer duration of residence. This pattern suggests barriers to access mental health care. Interventions that can lower these barriers are needed to enable newly settled refugees to access mental health care on equal terms with the native population.

## Background

Refugees have made up a large share of the immigrants to Scandinavia in recent decades. This is particularly true for Sweden, which since 1980 has received about 560 000 refugees and their families, primarily young adults and their children [1]. Pre-migration stress associated with war and political persecution in the country of origin, and long periods of uncertainty during the asylum process constitute potent risk factors for the development of mental health problems in refugees [2]. A systematic review of mental health in refugees, including studies of 7000 refugees resettled in Western countries, estimated the prevalence of Post-traumatic stress disorder (PTSD) to 8-10%, or about ten times the rate in the general population [3]. Other studies have additionally found elevated levels of depression [4,5]. In a Danish register-based study, refugees were found to have higher risk of having a first-time psychiatric contact for psychotic, affective and nervous disorders, compared with the native population [6]. Longitudinal studies of mental health in refugees showed slowly decreasing levels of psychiatric problems over time [7,8], albeit levels remained on a higher level than the majority population for as long as two decades after resettlement [9].

Despite this higher level of mental health problems, studies on utilization of mental health care service among refugees and immigrants showed lower use compared with natives, as well as different utilization patterns [10]. Studies on the use of somatic health care services among refugees and immigrants showed that service utilization increased with length of stay in the country and became more similar to the pattern of the majority population [11]. This suggests that newly arrived refugees and immigrants are confronted with barriers when accessing health care related to both structural and individual factors including organization of services as well as language and socio-cultural dimensions, which are gradually overcome with the process of adaptation to the new society.

Studies on use of psychotropic drugs in refugees and immigrants are rare in the literature [12-16]. Some register-based studies applied psychotropic medication as a proxy for mental health [14], while others also investigated access to treatment [15]. In a Swedish study on psychotropic drug use among 18-years old adolescents, descendants of immigrant women were found to have a lower use of medication, than the offspring of native Swedish women, despite assumed higher needs. The differences were most pronounced regarding the offspring to women from low-income countries, which was also the immigrant group with shortest time spent in Sweden [15]. Studies on antidepressant treatment showed that minority groups were at increased risk of not receiving adequate pharmacological therapy for depression compared to majority population, with immigrants presenting higher risk of not initiate treatment after having a prescription fulfilled [17], as well as of early discontinuation of treatment [18]. However, there were also studies reporting higher psychotropic drug use among immigrants compared with natives [12-14,16]. In a Swedish survey of immigrants from five different countries 5–15 years after settlement, for instance, immigrants generally reported a higher psychotropic drug use compared with Swedish-born. The difference regarding antidepressant was accounted for by a higher morbidity among the immigrants [12], while the higher use of anxiolytics and hypnotics seemed to be a result of differences in prescription patterns of doctors to immigrant patients compared with native patients, rather than a higher morbidity [13]. Although time in the new country has shown to be important when studying access to health care services [11], as well as immigrant and refugee mental health [7-9], no study on psychotropic medication among immigrants did focus specifically on the time aspect, or on psychotropic drug use in newly arrived refugees.

Considering that migration is an ongoing process with time as an important factor for both health and health care utilization, the aim of this study was to investigate the association between duration of residence and psychotropic drug use among refugees and their families who had been residents in Sweden during 1–10 years. We also studied whether psychotropic drug use in refugees differed by gender and region of origin.

## Method

This study was a cross-sectional, register study based on data from the Prescribed Drug Register held by the Swedish National Board of Health and Welfare and the Register of the Total Population held by Statistics Sweden. Data from the two registers was linked through the unique personal identification number assigned to each individual at birth or on obtaining residence permission in Sweden. The study was carried out with ethical approval from the Regional Ethical Committee in Stockholm.

### Study population

The index population was defined as all individuals born between 1973 and 1985, who immigrated to Sweden between 1998 and 2008 (thus with an age at immigration of 13–34 years) from Iraq, Iran, Eritrea, Ethiopia, Somalia or Afghanistan (n = 43 403), who remained as residents in Sweden in 2008. All Swedish-born residents, including descendents to immigrants, in these birth cohorts were included as a comparison population (n = 1 144 075). International adoptees (n = 60) and individuals missing information on domicile (n = 3 029) were excluded. The countries of birth of the immigrants included in the study all have a history of war, political turmoil and/or oppression and consisted of the large majority of refugees granted asylum during these years [19], which makes it reasonable to assume that with few exceptions the individuals in the study population were refugees or relatives to refugees.

### Outcomes

Information on use of psychotropic medication was obtained from the Swedish Prescribed Drug Register during 2009, which holds information on all drug prescriptions dispensed from Swedish pharmacies, excluding medical drugs consumed in inpatient hospital care. The outcome was defined as at least one dispensed, prescribed psychotropic drug during 2009 in the pharmaceutical ATC categories: a) neuroleptics (N05A), b) antidepressants (N06A), c) anxiolytics/hypnotics (N05B and N05C) or d) any psychotropic drug (at least one of the three categories). Anxiolytics and hypnotics were analyzed together as there is a broad overlap in the conditions for which they are used.

### Co-variates

Information on date of birth, gender, country of origin, year of immigration and domicile was obtained from the Register of the Total Population. Year of immigration was defined as the year of the last time the individual received residence permit for a minimum of one year in Sweden. Duration of residence was defined as the difference between the year of immigration and the year of study (2009). Domicile was categorized as metropolitan (Stockholm, Göteborg, Malmö), other city, and rural, based on the characteristics of the municipality.

### Statistical analysis

Logistic regression models were used to investigate the association between time in Sweden and psychotropic drug use. The outcomes were four binary variables (dispensing of drugs or not in 2009 in the categories described above). Due to the sample size, duration of residence was divided into three categories (0–3, 4–6, 7–10 years), considering the pattern of a non-linear increase in prevalence of psychotropic drug use with duration of residence evident from non-adjusted data. Based on their geographical location and economic characteristics, the regions of origin were grouped into Iraq/Iran, the Horn of Africa and Afghanistan. In a first analysis, a gender stratified logistic regression model was used to analyze the association between any dispensed psychotropic drug and duration of residence in three refugee study groups with the Swedish-born population as a reference category, adjusting for age and domicile.

In a second analysis, to investigate the association between duration of residence and specific psychotropic drug use, one logistic regression model was performed for each of the four categories of psychotropic drugs in the refugee population, with refugees from Iraq/Iran as the reference group, adjusting for age, gender, duration of residence and domicile. Age was introduced as a continuous variable in both analyses since there was a linear relation between age and all outcomes.

An interaction analysis of gender in the three refugee groups was made in a logistic regression model of having at least one dispensed psychotropic drug relative to the Swedish comparison group, with adjustment for age and domicile.

Statistical analyzes were performed using SPSS Statistics 20.

## Results

Table 1 shows demographic characteristics of the study population. Iraqis constituted more than half of the refugee group. Women formed the majority in the refugee groups from the Horn of Africa and Iran, while the opposite was true for Iraq and Afghanistan. There were small differences in mean duration of residence and also in age between the different nationalities. Age was also evenly distributed in relation to duration of residence, with refugees who had been 0–3 years in Sweden being less than one year younger than those who had been 7–10 years in Sweden (results not shown in table).

**Table 1:** **Demographic characteristics of the refugee population and the Swedish-born comparison group**

**Country of birth**	**N**	**Male sex (%)**	**Mean age (years)**	**Mean duration of residence (years)**
Iraq	24 885	55.0	28.9	3.8
Iran	6 476	43.6	28.8	3.6
Eritrea	1 780	39.4	30.0	2.5
Ethiopia	1 620	40.9	29.4	3.2
Somalia	5 811	43.1	28.3	3.0
Afghanistan	2 831	56.5	29.9	4.2
Sweden	1 144 075	51.7	29.1	-

Table 2 presents percentages of the four outcomes in 2009 by country of birth and duration of residence. The rates of having dispensed at least one psychotropic drug varied from 3.7% in the male group with the lowest rates (Eritrea) to 7.7% in male refugees from Afghanistan and the Swedish-born comparison population and from 3.6% (Ethiopia) to 12.6% (Afghanistan) in the female refugees.

**Table 2:** **Percentage of psychotropic drug use in 2009 by country of birth and duration of residence**

	**Men N**	**Women N**	**Any drug**		**Neuroleptics**		**Anti-depressants**		**Anxiolytics/hypnotics**	
			**M (%)**	**W (%)**	**M (%)**	**W (%)**	**M (%)**	**W (%)**	**M (%)**	**W (%)**
*Country of birth*										
Iraq	13 699	11 186	7.1	7.2	1.2	0.7	4.0	4.3	5.4	5.4
Iran	2 824	3 652	7.4	9.6	1.0	0.8	4.2	6.2	5.0	6.4
Eritrea	701	1 079	3.7	5.0	0.9	0.4	2.4	2.0	2.3	3.7
Ethiopia	662	958	4.0	3.6	0.9	1.0	2.1	1.8	2.7	2.7
Somalia	2 504	3 307	4.5	5.6	1.1	0.8	4.4	1.5	3.3	4.4
Afghanistan	1 600	1 231	7.7	12.6	1.2	1.2	4.4	8.5	5.3	7.9
Sweden	590 505	550 573	7.7	12.5	1.1	1.1	5.2	9.3	4.6	7.0
*Duration of residence*										
0-3 years	13 960	11 568	6.1	6.2	0.9	0.6	3.2	3.4	4.7	4.7
4-6 years	3 378	4 687	7.7	8.4	1.5	1.0	4.2	5.1	5.0	5.8
7-10 years	4 652	5 158	7.7	9.1	1.5	0.9	4.5	5.2	5.3	6.4

**Figure 1:**
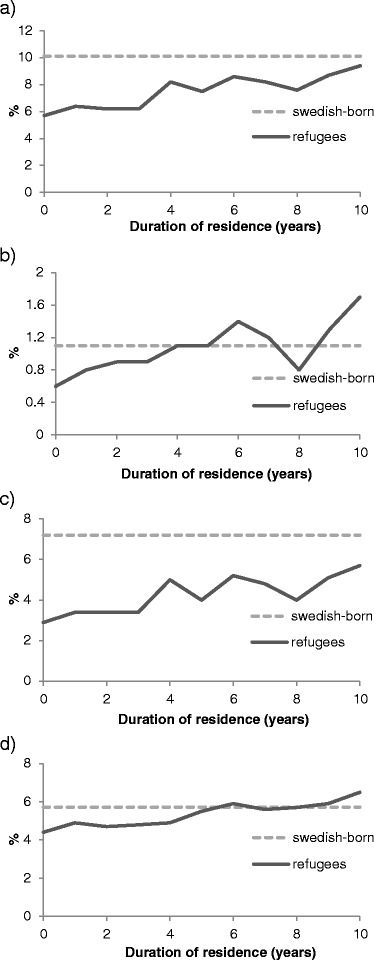
**Percentage of psychotropic drug use in refugees compared with Swedish-born. a)** Any drug **b)** Neuroleptics **c)** Antidepressants **d)** Anxiolytics/hypnotics.

Figure 1 demonstrates the rates of dispensed prescribed drugs in 2009 among refugees by duration of residence (1–10 years). Refugees with a duration of residence of 10 years had a similar rate of having at least one dispensed psychotropic drug compared with the Swedish-born. This pattern was relevant for neuroleptic drugs, as well as for anxiolytics/hypnotics, while levels of antidepressants remained lower also in refugees with 10 years of residency.

Table 3 presents gender stratified odds ratios (ORs) for having any dispensed psychotropic drug in 2009 by duration of residence and refugee group, compared with the Swedish-born population. Refugee men from Iraq/Iran, with a short duration of residence (0–3 years) had slightly lower ORs compared to the Swedish-born men 0.83 (95% CI 0.77-0.90), while for refugee men from the Horn of Africa, ORs were much lower for men with 0–3 years; 0.50 (95% CI 0.42-0.61), as well as 4–6 years of residence; 0.44 (0.28-0.68). For refugee women from Iraq/Iran and the Horn of Africa ORs were considerably lower than among the Swedish comparison group for all durations of residence, with a tendency to increase with longer duration; from 0.48 (95% CI 0.44-0.53) for 0–3 years to 0.72 (95% CI 0.65-0.80) for 7–10 years for women from Iraq/Iran and 0.36 (95% CI 0.30-0.41) to 0.50 (95% CI 0.39-0.66), respectively for women from the Horn of Africa. The refugees from Afghanistan had more similar adjusted ORs compared with the Swedish comparison population with no consistent trends by duration of residence. Refugee-women from Iraq/Iran and the Horn of Africa had lower adjusted ORs than men compared with the same gender in the Swedish comparison population. This gender pattern was confirmed in an interaction analysis, which showed a lower consumption of psychotropic drugs in women from Iraq/Iran and the Horn of Africa relative to men in comparison with Swedish-born of the same gender (p < 0.01)

**Table 3:** **Psychotropic drug use among refugees (n = 43 403) and Swedish-born (n = 1 144 075)**

		**Any drug**	
		**Men OR (95% CI)**	**Women OR (95% CI)**
*Region of origin and duration of residence*			
Sweden		1	1
Iraq and Iran	0-3 years	0.83 (0.77-0.90)	0.48 (0.44-0.53)
	4-6 years	1.03 (0.89-1.19)	0.69 (0.61-0.78)
	7-10 years	1.04 (0.93-1.17)	0.72 (0.65-0.80)
Horn of Africa	0-3 years	0.50 (0.42-0.61)	0.36 (0.30-0.41)
	4-6 years	0.44 (0.28-0.68)	0.35 (0.26-0.46)
	7-10 years	0.84 (0.57-1.23)	0.50 (0.39-0.66)
Afghanistan	0-3 years	0.96 (0.74-1.25)	1.19 (0.92-1.53)
	4-6 years	1.58 (1.14-2.18)	1.03 (0.76-1.38)
	7-10 years	0.67 (0.43-1.05)	0.86 (0.61-1.21)
*Age (years)*		1.03 (1.03-1.03)	1.03 (1.03-1.03)
*Domicile*			
Metropolitan		1	1
Other city		0.95 (0.93-0.97)	0.99 (0.98-1.01)
Rural		0.91 (0.88-0.93)	0.97 (0.95-1.00)

Table 4 shows a refugee specific logistic regression analysis in both genders of different types of psychotropic drugs with Iraq/Iran as the comparison group, adjusting for duration of residence. For neuroleptic drugs there were no differences between the refugee study groups, while for antidepressants the adjusted ORs differed greatly from 0.36 (95% CI 0.31-0.43) for refugees from the Horn of Africa and 1.49 (95% CI 1.16-1.76) for refugees from Afghanistan. The pattern was similar for anxiolytic/hypnotic drugs with adjusted ORs of 0.63 (95% CI 0.56-0.71) and 1.23 (95% CI 1.05-1.44) respectively.

**Table 4:** **Psychotropic drug use among refugees by duration of residence, gender, age, country of birth and domicile**

	**Any drug OR (95% CI)**	**Neuroleptics OR (95% CI)**	**Antidepressants OR (95% CI)**	**Anxiolytics/hypnotics OR (95% CI)**
*Duration of residence*				
0-3 years	1	1	1	1
4-6 years	1.25 (1.13-1.37)	1.54 (1.20-1.97)	1.31 (1.15-1.48)	1.09 (0.97-1.22)
7-10 years	1.28 (1.17-1.40)	1.50 (1.19-1.89)	1.30 (1.15-1.46)	1.15 (1.03-1.27)
*Gender*				
Male	1	1	1	1
Female	1.13 (1.05-1.22)	0.66 (0.54-0.81)	1.21 (1.10-1.34)	1.12 (1.03-1.22)
*Age*	1.06 (1.05-1.08)	1.05 (1.02-1.08)	1.08 (1.06-1.09)	1.06 (1.05-1.07)
*Region of origin*				
Iraq and Iran	1	1	1	1
Horn of Africa	0.63 (0.56-0.70)	0.96 (0.75-1.23)	0.36 (0.31-0.43)	0.63 (0.56-0.71)
Afghanistan	1.40 (1.22-1.60)	1.19 (0.83-1.71)	1.49 (1.16-1.76)	1.23 (1.05-1.44)
*Domicile*				
Metropolitan	1	1	1	1
Other city	0.88 (0.81-0.95)	0.73 (0.59-0.90)	0.86 (0.78-0.96)	0.84 (0.77-0.92)
Rural	1.20 (1.02-1.41)	0.81 (0.49-1.33)	1.21 (0.98-1.51)	1.08 (0.88-1.31)

## Discussion

In this study of psychotropic medication in a national cohort of more than 40 000 young adult refugees and their families in Sweden, we found lower rates of dispensed psychotropic drugs among recently settled refugees, compared to Swedish-born, with an increase with duration of residence. The adjusted ORs for having dispensed at least one psychotropic drug during 2009 were lower for refugees from Iraq/Iran and the Horn of Africa who had resided for 0–3 years compared with native Swedish residents, while the adjusted ORs were more similar for those with 7–10 years of residency. Afghans had more similar ORs compared with the Swedish-born, and refugee women generally had lower ORs compared with refugee men relative to the native Swedish residents. Refugees from the Horn of Africa presented lower adjusted ORs of antidepressants and axiolytics/hypnotics compared with the other refugee groups, while the use of neuroleptic drugs was similar between refugee groups.

It is reasonable to believe that levels of drug consumption may reflect both mental health status as well as access to care. As mentioned above, high levels of PTSD and depression have been consistently reported in refugee populations [3-6]. This is true also for recent studies of the newly settled refugees in the population in focus in this study [5,20,21]. In a study on 366 newly settled Iraqi refugees (age 18–84 years old) to the United States 18–36 months post-arrival the prevalence of depression was estimated to 50%, and 31% of participants were identified as at risk for PTSD [5]. A study on asylum-seekers and refugees from Afghanistan, Iran and Somalia (mean age 37 years) with on average 5.6 years since arrival in the Netherlands, reported symptoms of PTSD in 28.1% of asylum-seekers and 10.6% of refugees, and symptoms of depression/anxiety in 68.1 and 39.4% respectively [21]. The highest rate of PTSD-symptoms was found in Iranian asylum-seekers (43.4%), and the lowest rate in Afghan and Somali refugees (6.0 and 4.0% respectively). For depression and anxiety symptoms, again, the lowest rates were found in Afghan and Somali refugees (28.9 and 16.7% respectively). A study on 180 Somali refugees (mean age 40.4 years) who had been living in the United Kingdom for an average of 8 years, reported a rate of depression and/or anxiety-symptoms of 25%, with the recently arrived refugees presenting the highest levels of disorder [20]. Thus, it seems most likely that the rates of medication found in the refugees in this study are low in relation to their burden of mental ill-health. The time pattern found in the studies mentioned above [7-9], with the highest rates of mental disorder in the recently settled refugees, contrasts with the low rates of dispensed drugs during these years in our study. Thus it seems reasonable to interpret the low rates of dispensed drugs in our study as being more dependent on a lower access to care than on a better health status.

There are both formal and informal barriers to health care that might be relevant to understand this pattern [22]. Formal barriers refer to organizational aspects of the healthcare system. In Sweden, all individuals with a residence permit are entitled to universal health care on equal terms. According to Swedish law, asylum-seekers and newly arrived refugees should be offered a health examination of physical and mental health, as well as information about the Swedish health system [23], however, only 54% of asylum-seekers underwent the examination in 2011 [24]. For newly arrived refugees and family members who did not go through the asylum reception system the figure was even lower. Moreover, although Swedish health care are mainly tax-financed, there are elements of user payment that may constitute barriers to people with very low income levels, such as newly settled refugees. In 2009 patients paid the whole cost for drugs up to 900 SEK/year (≈88 EURO), thereafter purchase was partially subsidized up to a maximum of 1800 SEK/year (≈176 EURO), after which all costs were paid by the county council [25]. The eventual effect of co-payment for utilization is difficult to estimate, but Danish studies have shown that costs may have some influence of drug use among economically weak groups [26]. Informal barriers include aspects such as knowledge about the health system, language skills and socio-cultural factors affecting the self-perceived needs and health seeking-behavior [10,11,27]. Language barriers constitute a major obstacle for newly settled refugees in contacts with the health care system [27,28]. Access to professional interpreters with training in medical terminology is essential for effective communication, however lack of skilled interpreters and organizational problems restrict its use, compromising the delivery of quality care [28]. In addition to language, communication is also affected by cultural differences, as culture influences the concepts and explanatory models surrounding health and illness and the way in which mental health problems are expressed [10,27,29]. Acculturation, the process of integration of concepts and beliefs from the new society, is thus an important aspect of care-seeking patterns for mental health problems in refugees [10]. As pointed out by Ingleby [27], attitudes towards mental health among non-western immigrants are usually constructed in a context in the country of origin where few services are available and the concept of mental illness often is limited to extreme disturbances and associated with considerable stigma. Lack of cultural awareness among health care professionals may affect the interpretation of symptoms and thereby delay or compromise the quality of diagnosis and treatment [15,30]. The results of the study on Somali refugees in the UK made by Bhui et al. [20], mentioned above, suggests that there may be important differences in barriers to care between Sweden and the UK for refugees. In the UK study approximately 14% of the Somali refugees were taking psychotropic medication, a considerably higher number than the rates of psychotropic medication found in our data (4.5 and 5.6% in the Somali men and women respectively). Thus, comparative studies between refugees in different societies are indicated to shed further light on the barriers to care for refugees.

In refugee populations mental health is affected by pre- and post-migration factors. The relative influence of the pre-migration traumatic experiences and exile-related factors may change during the migration process [31], although some studies stressed that the negative impact of severe past trauma tended to persist over time [8]. Some studies also stressed the role of post-migration factors in moderating the effect of pre-migration trauma, where a continued stress in exile may prevent the process of recovery from psychological problems, thus maintaining a high load of mental ill-health over time [31].

A striking feature of the results was the variation in dispensed prescriptions by region of origin, with refugees from the Horn of Africa showing significantly decreased ORs of dispensed, prescribed antidepressants and anxiolytics/hypnotics, compared with refugees from Iraq/Iran, while levels were elevated among Afghans. This pattern to a certain extent may reflect differences in prevalence of psychiatric disorders, since a similar pattern was observed in studies among asylum-seekers and refugees from Iran, Afghanistan and Somalia in the Netherlands, where Somalis were found to have lower rates of PTSD and depression/anxiety symptoms, as well as having a lower use of mental health services and psychotropic drug, compared with other refugee populations [32]. Another possibility is that these differences reflect different manners of expressing mental health problems in different cultural contexts, rather than differences in disorders per se [27]. Afghan men and women differed from the other refugee groups, by presenting similar levels of psychotropic drug use as the Swedish-born population already during the first years after settlement, however, the study population was too small to allow for any firm conclusion for the time pattern of drug consumption for this refugee group.

The use of neuroleptic drugs was similar between refugee groups. One explanation for this may be that neuroleptics are prescribed for more severe conditions, often involving psychotic symptoms, where the need for psychiatric treatment is more obvious, and often initiated by other people than the patient him/herself. Refugee women are generally found to present higher prevalence of depression, as well as PTSD [21,33]. Thus, the relatively low levels of psychotropic drug use among refugee women from the Horn of Africa and Iraq/Iran found in this study are noteworthy and indicate that there may be additional barriers to care affecting female refugees. Language problems, because of less involvement with the surrounding society through employment, may be one such factor [10]. Another explanation might be a lack of recognition of the specific experiences of refugee women [34], with the consequence that the traumas and symptoms of mental illness among female refugees are overseen within the health care system.

### Strengths and limitations

The major strength of the study was that it employed national register data covering the entire Swedish population, avoiding the risk of biased results due to selective attrition and/or recall bias, which is a common problem in surveys of minority populations [13]. The large population sample made it possible to analyze time patterns across different outcomes and among groups with different origin.

Some studies in the literature have used psychotropic drug use as indicators of mental health problems in refugees and immigrants. The time dependent pattern of drug use in this study implies considerable caution in the interpretation of such studies, unless the relation between drug use and mental health has been investigated in that specific population in a longitudinal perspective.

The register-based design of this study comes with the inherent limitations of all such studies of relying on data which was collected for purposes other than the present research, which restricts the possible questions to be posed and conclusions drawn.

Firstly, since we did not have information about the actual mental health status of the study population, we cannot draw any final conclusions on the reason behind the increase in psychotropic medication with duration of residence. Thus, we cannot completely rule out the possibility that the increased drug use over time, besides an improved access, to some extent also reflects deteriorating mental health. Neither can we estimate to what extent the variation of drug use between refugee populations reflects differences in the prevalence of mental health problems rather than different patterns in care-seeking behavior and barriers to care. Studies with information on psychiatric disorders and drug use in refugees are needed to shed further light on these patterns.

Secondly, the Swedish registers lack reliable information on pre-migration factors regarding education and domicile (rural/urban), as well as reason for migration. In this study, country of origin was used as a proxy for refugee status, which means the study population may also include individuals migrating for other reasons than refugee, however, this number was assumed to be negligible. Another noteworthy shortcoming in the register information in this study is that the indicator of time after settlement in Sweden is based on the date when a residence permit was granted, and not when actually entering Sweden. As a result the asylum period was not included when we calculated duration of residence, which means that most individuals had resided in Sweden for a longer period than estimated. The average waiting time for asylum was about one year during the time of the study, which indicates that the true time lapse in access to care was even more protracted than shown by our results. The waiting time, however, was similar in the six refugee study groups. Thus, differences in waiting time cannot be expected to have introduced any major bias in our analysis of differences between study groups [35]. Another shortcoming, is that we only had information on the last time the individual received residence permit in Sweden. It seems most unlikely, however, that the re-immigration would be a common occurrence in these Non-European refugees, bearing in mind that many refugees come from countries that are still conflict-stricken and that this would necessitate a new asylum application in most cases. Finally, the Swedish Prescribed Drug Register only holds information on drugs purchased at Swedish pharmacies. It is not altogether unlikely that immigrants may have other ways of obtaining prescribed drugs, illegally, legally in other countries or through the internet. It seems quite unlikely, however, that this would explain the time-pattern of dispensed drugs in this study.

## Conclusion

Psychotropic drug use is sometimes used as an indicator of mental health problems in large populations in national prescribed drug registers. The time dependent pattern of psychotropic drug use in this study, and the large variation of drug use between different refugee population implies considerable caution in the interpretation of such studies, unless the relation between psychotropic medication and mental health has been investigated in that specific refugee population in a longitudinal perspective.

The present study showed a low use of psychotropic drugs during the first years after having received the permanent residency among newly settled refugees and their families compared with the Swedish-born population, suggesting barriers in access to adequate treatment. Psychiatric illness, such as depression and PTSD, are potentially disabling diseases and may hamper a successful integration into the new society. Further studies are needed to elucidate the barriers to mental health care for refugees so that effective measures to improve access to mental health services can be implemented.
